# Light Flux Density and Photoperiod Affect Growth and Secondary Metabolism in Fully Expanded Basil Plants

**DOI:** 10.3390/foods13142273

**Published:** 2024-07-18

**Authors:** Luigi d’Aquino, Rosaria Cozzolino, Livia Malorni, Thierry Bodhuin, Emilia Gambale, Maria Sighicelli, Brigida Della Mura, Cristina Matarazzo, Sonia Piacente, Paola Montoro

**Affiliations:** 1 Italian National Agency for New Technologies Energy and Sustainable Economic Development (ENEA), Portici Research Centre, Piazzale E. Fermi 1, 80055 Portici, Italy; emilia.gambale@enea.it; 2 Institute of Food Science, National Council of Research (CNR), Via Roma 64, 83100 Avellino, Italy; livia.malorni@isa.cnr.it (L.M.); cristina.matarazzo96@gmail.com (C.M.); 3 FOS S.p.A., Via E. Melen 77, 16152 Genova, Italy; thierry.bodhuin@fos.it; 4 Italian National Agency for New Technologies Energy and Sustainable Economic Development (ENEA), Casaccia Research Centre, Via Anguillarese 301, Santa Maria di Galeria, 00060 Roma, Italy; maria.sighicelli@enea.it; 5 Department of Science, University of Basilicata, Viale dell’Ateneo Lucano 10, 85100 Potenza, Italy; brigida.dellamura@unibas.it; 6 Department of Pharmacy, University of Salerno, Via Giovanni Paolo II, 84084 Fisciano, Italy; piacente@unisa.it (S.P.); pmontoro@unisa.it (P.M.)

**Keywords:** indoor farming, precision agriculture, LED lighting, plant metabolomics, volatile organic compounds

## Abstract

Indoor production of basil (*Ocimum basilicum* L.) is influenced by light spectrum, photosynthetic photon flux density (PPFD), and the photoperiod. To investigate the effects of different lighting on growth, chlorophyll content, and secondary metabolism, basil plants were grown from seedlings to fully expanded plants in microcosm devices under different light conditions: (a) white light at 250 and 380 μmol·m^−2^·s^−1^ under 16/8 h light/dark and (b) white light at 380 μmol·m^−2^·s^−1^ under 16/8 and 24/0 h light/dark. A higher yield was recorded under 380 μmol·m^−2^·s^−1^ compared to 250 μmol·m^−2^·s^−1^ (fresh and dry biomasses 260.6 ± 11.3 g vs. 144.9 ± 14.6 g and 34.1 ± 2.6 g vs. 13.2 ± 1.4 g, respectively), but not under longer photoperiods. No differences in plant height and chlorophyll content index were recorded, regardless of the PPFD level and photoperiod length. Almost the same volatile organic compounds (VOCs) were detected under the different lighting treatments, belonging to terpenes, aldehydes, alcohols, esters, and ketones. Linalool, eucalyptol, and eugenol were the main VOCs regardless of the lighting conditions. The multivariate data analysis showed a sharp separation of non-volatile metabolites in apical and middle leaves, but this was not related to different PPFD levels. Higher levels of sesquiterpenes and monoterpenes were detected in plants grown under 250 μmol·m^−2^·s^−1^ and 380 μmol·m^−2^·s^−1^, respectively. A low separation of non-volatile metabolites based on the photoperiod length and VOC overexpression under longer photoperiods were also highlighted.

## 1. Introduction

Indoor farming represents a promising approach to supply year-round vegetable produce in environments where conventional agricultural approaches cannot be applied, such as urban and extreme settings; this strategy could provide local communities with a new tool to face food security threats, particularly those deriving from climate change [1,2]. Suitable light supply is one of the major issues that should be addressed in indoor plant growing, and recent advancements in indoor farming technology are largely due to improvements in artificial lighting. The diffusion of light-emitting diode (LED) technology is probably the most relevant development in this field [3]. Thanks to the flexibility of LED technology, which enables researchers to supply plants with selected wavelengths, the possibility of driving plant growth and metabolism by managing lighting conditions has been demonstrated [4,5,6,7]. Different lighting conditions have been reported to affect the growth, development, and metabolic profiles, especially the essential oil profile, of basil (*Ocimum basilicum* L., Lamiaceae) [8,9,10,11,12,13,14,15,16,17,18,19,20,21,22,23]. Concerning the biological activities and nutritional potential of basil caffeic, vanillic, and rosmarinic acids, quercetin, rutin, apigenin, chlorogenic, and p-hydroxybenzoic have been recognized as antioxidant constituents. The essential oil of basil is reported to contain eugenol, chavicol, and terpenoids. Due to the presence of these specialized metabolites, basil is widely cultivated and used as both a vegetable and medicinal plant. The latter is justified by different activities reported in the literature (e.g., anti-cancer, radioprotective, anti-microbial, anti-inflammatory, immunomodulatory, anti-stress, anti-diabetic, anti-pyretic, anti-arthritic, and anti-oxidant properties) [24].

Despite the increasing number of papers available in the scientific literature, few studies have dealt with fully expanded plants, a common harvesting stage in market-oriented basil cultivations. In previous works, d’Aquino et al. [12,25] grew basil in an innovative microcosm device from the seedling to the flowering stages, in order to investigate the effects of environmental conditions on fully expanded plants, confirming that different light spectra differently affect biomass yield and metabolism in fully expanded basil. In those studies, white light was associated with higher yield compared to blue–red light when supplied at the same photosynthetic photon flux density (PPFD), and white and blue–red lights were associated with different phenolic profiles in the plants. In the present study, basil was grown in microcosm devices from the seedling to the flowering stage under white light at (a) two different PPFD levels under the same photoperiod and at (b) two different photoperiods under the same PPFD level, in order to test the effects of two white light regimes on plant growth, chlorophyll content index (CCI), and the production of volatile and non-volatile secondary metabolites.

## 2. Materials and Methods

### 2.1. Chemicals and Reagents

Ultra-pure water from a Milli-Q system (Millipore, Bedford, MA, USA) with a conductivity of 18 MΩ was used throughout the analytical procedures. Ethanol was from Romil (Cambridge, UK). Sodium sulfate and 2-octanone were from Sigma-Aldrich (St Louis, MO, USA). Helium at a purity of 99.999% (Rivoira, Milano, Italy) was used as the gas chromatography (GC) carrier. The glass vials and the SPME fibers were from Supelco (Bellefonte, PA, USA). The capillary GC-MS column HP-INNOWax (30 m × 0.25 mm × 0.5 μm) was from Agilent Technologies Inc. (Santa Clara, CA, USA). The SPME fibers were conditioned prior to their first use as recommended by the manufacturer but below the maximum recommended temperature. Before each analysis, the fibers were conditioned for 5 min at the GC injector port temperature, and the blank level was determined.

### 2.2. Germplasm and Growing Conditions

**Figure 1:**
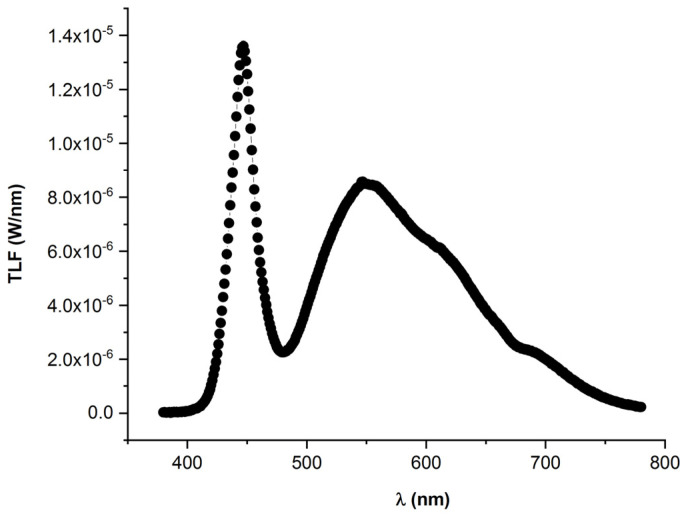
The light spectrum in the wavelength region λ 380 ÷ 780 nm was determined at about 80 cm from the light sources.

Plantlets of basil-type Genovese cv. “Bonsai” (Blumen Vegetable Seeds, Milano, Italy), at the stage of four true leaves, were transplanted into two different microcosms set up as described in the work by d’Aquino et al. [24]. Each microcosm was equipped with 6 cylindrical pots (60 cm height, 20 cm diameter), spaced 25 cm apart from each other, and each pot was filled with commercial potting soil (60% blond peat, 20% brown peat, 20% pumice 3–6 mm, pH 6.5). Three to six seedlings were jointly transplanted, to obtain a plant density typical for basil crops targeted to industrial processing. Temperature conditions were 20–26 °C (night-day) and 18–22 °C (night-day) in the epigeal and hypogeal chambers, respectively. A white light spectrum was obtained in each microcosm using LEDs LUXEON SunPlus 20 Cool White (Lumileds, Schiphol, The Netherlands) and Oslon^®^ SSL 80 Cool White (Osram Opto Semiconductor, Regensburg, Germany). The light spectrum in the wavelength region λ 350 ÷ 800 nm was determined at about 80 cm from the light source, i.e., at the seedlings level, using a spectroradiometer OL-770VIS (Gooch and Housego, Ilminster, UK) equipped with an OptoPolymer integrating sphere (Figure 1). The PPFD was determined with a LI-190R Quantum Sensor and a LI-1500 Light Sensor Logger (LI-COR Biosciences, Lincoln, NE, USA). In the first experiment (the ‘PPFD experiment’), aimed at testing the effects of two different PPFD levels, the plants were grown under light intensities of 250 and 380 μmol·m^−2^·s^−1^ (‘Microcosm 250’ and ‘Microcosm 380’, respectively), under the same photoperiods of 16/8 h light/dark. This experiment followed the same PPFD sequences, increasing (0, 25, 50, 75, and 100%) and decreasing (100, 75, 50, 25, and 0%) from dark to full light conditions. In a second experiment (the ‘photoperiod experiment’), aimed at testing the effects of two different photoperiods, the plants were grown under a light flux of 380 μmol·m^−2^·s^−1^ and two different photoperiods of 16/8 h and 24/0 h light/dark (‘Microcosm 16’ and ‘Microcosm 24’, respectively). In Microcosm 16, the PPFD sequences—increasing and decreasing in the transition from dark to full light conditions—were indicated for the PPFD experiment. In the PPFD experiment, the plants were watered with about 14.1 and 14.4 L of water/pot in Microcosm 250 and Microcosm 380, respectively, batching the total amount of water in the growing period, according to the biomass growth. In the photoperiod experiment, the plants were watered with about 10.9 and 11.9 L water/pot in Microcosm 16 and Microcosm 24, respectively, again batching the total amount of water during the cultivation, according to the plant growth. In the PPFD experiment, the aerial parts were harvested 50 days after transplant, i.e., when the plants had reached the full flowering stage, while in the photoperiod experiment, the aerial parts were harvested 36 days after transplant when the plants were at the beginning of the flowering stage.

### 2.3. Biometric Determinations

All the plants in each pot were considered as one replicate; therefore, the samples from each pot were jointly collected and the biometric determinations were carried out on a total of six groups of plants per light treatment. At the harvest stage, the plant heights were recorded and leaves, stems and inflorescence axes were independently collected to determine fresh and dry weights. The data were analyzed by one-way ANOVA using the SPSS 27 software package (www.ibm.com/software/analytics/spss, accessed on 20 November 2022).

### 2.4. Chlorophyll Concentration Index (CCI) Determination

The CCI was determined on six middle and apical leaves per pot using the chlorophyll concentration meter Model MC-100 (Apogee Instruments, Logan, UT, USA). The determinations were carried out 48 days after the transplant in Microcosms 250 and 380, and 32 days after the transplant in Microcosms 16 and 24, i.e., on fully expanded plants at the beginning of the flowering stage. The data were analyzed by one-way ANOVA using the software package reported above.

### 2.5. Liquid Chromatography–Electrospray Ionization–Linear Ion Trap Quadrupole–Orbitrap–Mass Spectrometry (LC-ESI-LTQ-Orbitrap-MS) Analysis

Fully expanded leaves were collected from the middle and apical parts of the plants at the end of the cultivation period in both the PPFD and photoperiod experiments, i.e., 49 days after the transplant for Microcosms 250 and 380 and 35 days after the transplant for Microcosms 16 and 24. Samples were independently collected from each pot, generating six independent samples from each microcosm (12 samples for each experiment). The leaves were pulverized in a mortar immediately after collection using liquid nitrogen and stored at −80 °C until further processing. A 150 mg aliquot of each sample was homogenized with 2 mL of ethanol:water (1:1). The extract was sonicated for 10 min and centrifuged at 3000 rpm in a MiniSpin plus Centrifuge (Eppendorf, Hamburg, Germany) for 10 min to remove coarse residues. The supernatant was dried under a nitrogen stream to remove the solvent and dissolved in 2 mL of methanol. To remove chlorophyll, a 1 mL aliquot was loaded onto a solid-phase extraction cartridge Strata^®^ SCX 55 μm, 70 Å (Phenomenex, Torrance, CA, USA), previously conditioned with methanol, and the sample was eluted with 1 mL of methanol. The eluted samples were dried under a nitrogen stream to remove solvent and dissolved in methanol:water (1:1) (LC-MS grade) at a final concentration of 1 mg/mL. Aliquots of 10 μL were injected in an Accela HPLC System coupled with a hybrid mass spectrometer, combining a linear quadrupole trap (LTQ) and an Orbitrap mass analyzer equipped with an electrospray (ESI) source. Chromatographic separation was performed on an HSS T3 column of 100 × 1.0 mm, with a particle size of 1.8 m (Waters, Milford, MA, USA), with the following binary gradient at 150 μL/min: 0–30 min, linear from 5 to 95% B; 30–35 min, isocratic 95% B; 36–42 min, isocratic 5% B. The parameters of the ESI source were as follows: capillary voltage—48 V, lens voltage—176.47, capillary temperature 280 °C, sheath and auxiliary gas flow (nitrogen) 15 and 5, gas sweep 0, spray voltage 5. The mass range used for acquisition was 120–1600 *m*/*z*. For the fragmentation studies, a data-dependent experiment was performed, selecting ionic precursors corresponding to the most intense peaks obtained during LC-MS acquisition. Instrumental control, data acquisition, and data analysis were performed using the Xcalibur version 2.1 program. Multivariate analysis of the data was carried out with SIMCA®-P+ 12.0 software (Umetrics, Umeå, Sweden), which models each class separately through a synthetic representation provided by the significant principal components present in each class. Before the multivariate analysis, data were aligned with mz-Mine 3 software and logarithmically transformed and scaled using Pareto scaling as the mode. Principal component analysis (PCA) was used as a projection method to compare the different samples.

### 2.6. Sample Preparation and Headspace Solid Phase Microextraction (SPME) Procedure

Leaf sampling was conducted as reported above. The samples were stored at −80 °C using headspace solid-phase microextraction combined with gas chromatography-mass spectrometry (HS-SPME/GC-MS). Volatile profiling was carried out as described in the work by Cozzolino et al. [26], using a conditioned 2-cm long DVB/CAR/PDMS (50/30 µm) fiber with an extraction temperature and time of 40 °C and 20 min, respectively. Aliquots made up of 0.75 g of leaf tissues were introduced into a 20 mL headspace vial with a screw cap (Supelco, Bellefonte, PA, USA) containing 5 mL of ethanol 5% (*v*/*v*), 0.5 g of sodium sulfate, and 12.5 μL from a stock solution of 2-octanone 25 ppb, used as an internal standard (IS). After stirring, vials were immediately sealed and placed with a Teflon (PTFE) septum and an aluminum cap (Chromacol, Hertfordshire, UK), and set at 40 °C for 20 min to equilibrate the system. Extraction and injection were carried out using an autosampler MPS 2 (Gerstel, Mülheim, Germany). To absorb volatiles on the fiber surface, the fiber was finally introduced in the vial’s septum for 20 min.

### 2.7. Gas Chromatography—Quadrupole Mass Spectrometry (GC-qMS) Analysis

The HS-SPME fiber was introduced and maintained for 10 min in the injector of a GC 7890A gas chromatography system, coupled with an Agilent 5975 C mass spectrometer. Volatile organic compounds (VOCs) were separated by a capillary column HP-INNOWax (30 m × 0.25 mm × 0.5 µm) (Agilent, Santa Clara, CA, USA) using helium as a carrier gas at 1.5 mL·min^−1^. At the beginning of the data acquisition, the temperature was set at 40 °C for 5 min, ramped up to 240 °C at 4 °C·min^−1^, and held constant at 240 °C for 5 min. The transfer line, ion source, and quadrupole temperature were 240, 230, and 150 °C, respectively. A pulsed splitless mode was used for the analysis. Mass spectra were acquired at an ionization energy of 70 eV and volatile components were detected using a mass selective detector operating in a mass range between *m*/*z* 30 and 300 at a 2.7 scans·s^−1^ rate. Each analysis was performed in duplicate in a randomized sequence, which included blank runs. Metabolite identification was based on mass spectra matching with the database library (NIST, version 2005; Wiley, Hoboken, NJ, USA, version 2007) and comparing the retention times with a reference library created from analytical standards. Furthermore, the identification of VOCs was carried out by matching the retention indices (RIs) (as Kovats indices) with those in the literature data [27]; this was determined relative to the retention times of n-alkanes (C_8_-C_20_) using linear interpolation, compared with those of the literature data or authentic compounds. Single VOC areas were measured from the total ion chromatogram. The relative peak area (RPA%) for each metabolite was automatically adjusted according to the IS peak area.

### 2.8. Multivariate Data Analysis

For data visualization by PCA, a pseudo-targeted approach was followed. First, a matrix was obtained using the LC-ESI-MS peak areas corresponding to the compounds identified by MS and MS/MS data, extracted from the LC/ESI/MS profile chromatograms obtained in the negative ion mode. After exporting the treated data in a table format, a multivariate statistical analysis of the data matrix was performed using SIMCA®-P+ 12.0 software via the PCA method. Pareto scaling was used to normalize the data before multi-variate data analysis. A pseudo-targeted approach was followed as indicated for GC-MS data analysis. A matrix was obtained using the GC-MS peak areas corresponding to the compounds identified and, after exporting the treated data in a tabular format, multivariate statistical analysis of the data matrix was performed using SIMCA®-P+ 12.0 software through the PCA method. Pareto scaling was used to normalize. The models obtained were validated using transversal analysis techniques and permutation tests, in accordance with the new standardization practices, to minimize falsehoods and obtain robust statistical models [27]. A single dataset containing GC-MS and LC-MS data from all identified compounds in the samples from plants grown in Microcosms 250 and 380 was prepared for multivariate data analysis. The original GC-MS matrix was made using 70 variables and 48 observations; the original LC-MS matrix was made using 44 variables and 48 observations. The final matrix—realized by data fusion—was represented by 114 variables and 48 observations. The GC-MS and LC-MS signals, which were the areas under the curve obtained by the two tools, were normalized by Pareto scaling to balance them. The PCA approach was applied to the final dataset using SIMCA®-P+ 12.0 software. The same approach described above was applied to obtain GC-MS and LC-MS data fusion of all identified compounds in the samples of plants grown under different photoperiods. The models obtained were validated using transversal analysis techniques and permutation tests, in accordance with the new standardization practices, to minimize falsehoods and obtain robust statistical models [28]. The combination of variables corresponding to the non-volatile metabolites with those relating to the volatile compounds in a single plot following a ‘low-level’ data fusion approach [29] was performed. After targeted analysis of the profiles of volatile and non-volatile compounds, a comprehensive metabolomics fingerprint was constructed by means of a data fusion strategy. This involved combining data from different instrumental measurements. The data fusion strategy consisted of merging the individual LC-MS and HS-SPME/GC-MS data matrices and concentrating the resulting data into a single final matrix. The HS-SPME/GC-MS data were treated by the MetaboAnalyst 5.0 web-based tool (Xia Lab, McGill University, Montreal, QC, Canada). In brief, the raw data were subjected to the IS ratio correction, sample median, and data scaling by autoscaling. The n univariate statistical analysis (ANOVA, *p* < 0.05) was performed to evaluate significant statistical differences among the VOC concentrations in each sample group (*p* < 0.05; Supplementary Tables S1 and S2).

## 3. Results

### 3.1. Biometric Determinations

**Figure 2:**
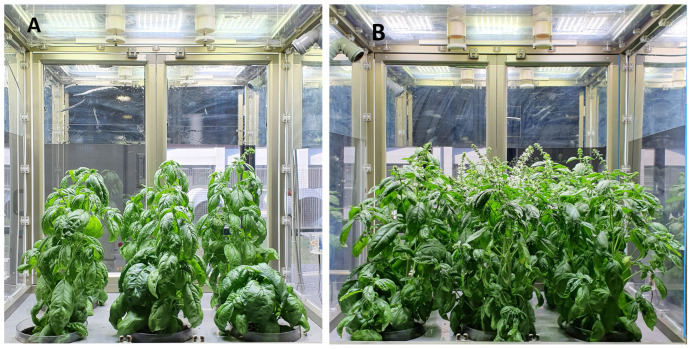
Basil plants at the end of the cultivation period in the microcosms under white light at 250 (**A**) and 380 (**B**) μmol·m^−2^·s^−1^.

**Figure 3:**
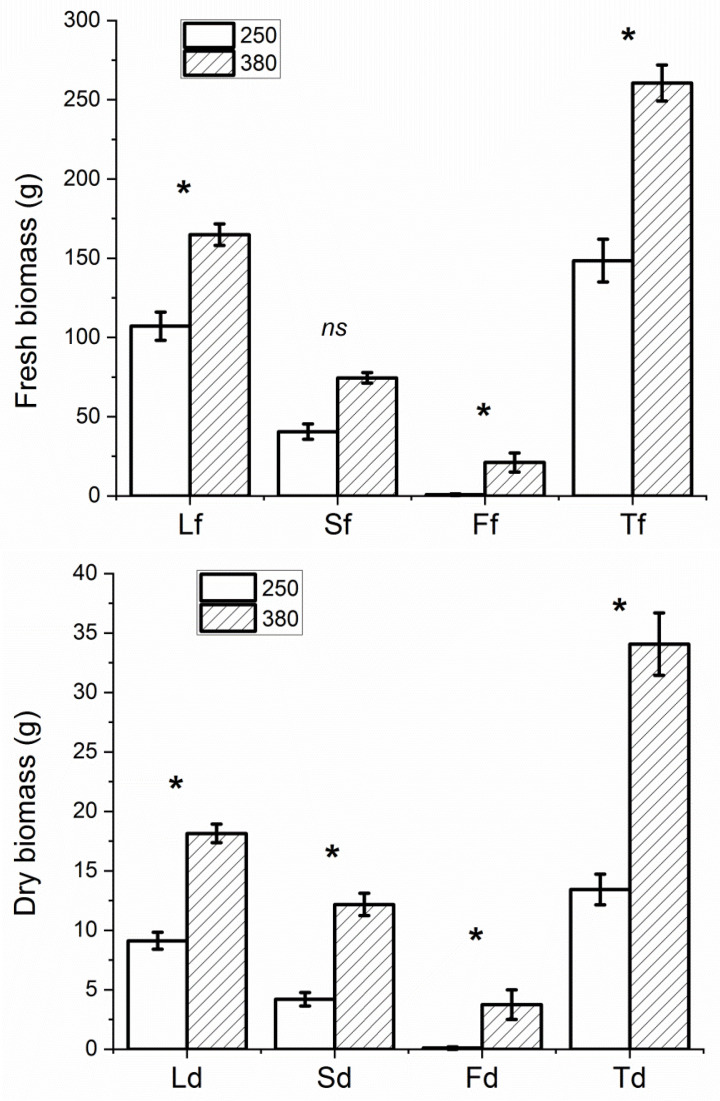
Average fresh (f, **upper**) and dry (d, **lower**) weights of leaves (L), stems (S), and inflorescence axes (F), and average weights of the total aerial biomasses (T) of plants in Microcosms 250 (‘250’) and 380 (‘380’) determined at harvest time. Bars indicate the mean values and standard errors (*n* = 6 replicates). Non-significant and significant differences at *p* ≤ 0.05 are indicated as ns and *.

**Figure 4:**
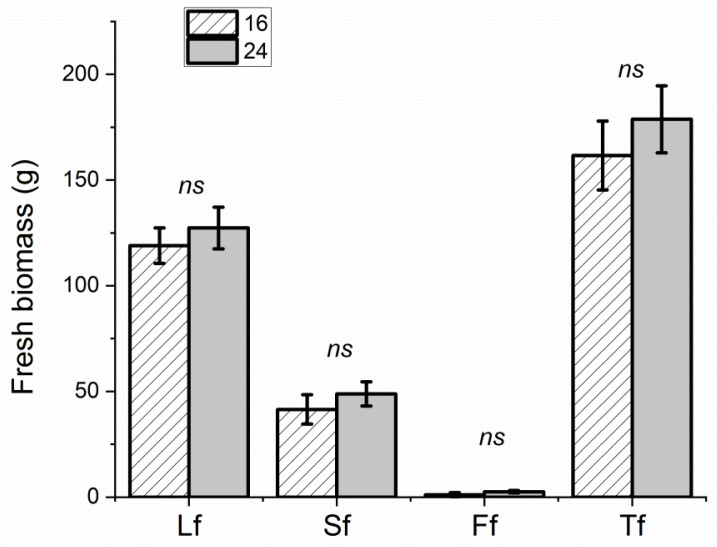

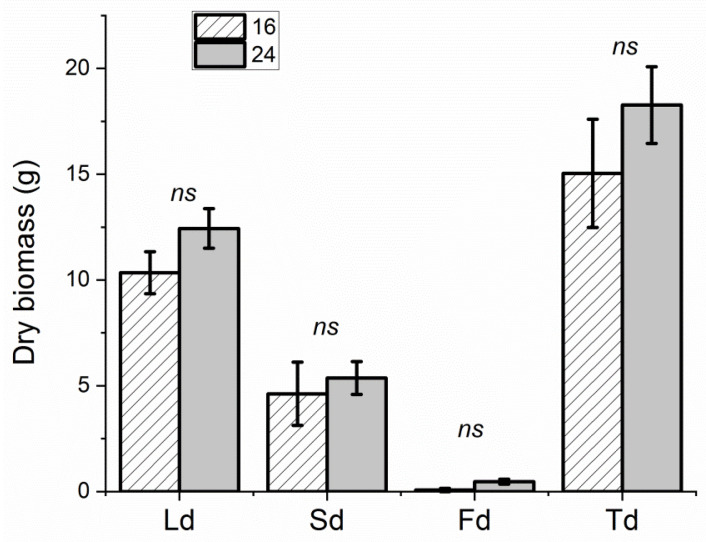
Average fresh (f, **upper**) and dry (d, **lower**) weights of leaves (L), stems (S), and flowers (F), and average weights of the total aerial biomasses (T) of plants in Microcosms 16 (‘16’) and 24 (‘24’) determined at harvest time. Bars indicate the mean values and the standard errors (*n* = 6 replicates). Non-significant differences are indicated as ns.

No differences in phenotypical and phenological development were observed in plants under different PPFD levels and photoperiods. Different plant heights and yields were recorded in the two experiments, due to the different durations of the cultivation periods, but no significant differences in plant heights were scored at harvesting time in the PPFD experiment (with average heights of 43.6 ± 5.4 cm and 47.9 ± 2.1 cm in Microcosms 250 and 380, respectively) and the photoperiod experiment (21.2 ± 2.5 cm and 22.3 ± 1.5 cm in Microcosms 16 and 24, respectively). A higher yield was recorded in plants grown in Microcosm 380 compared to those grown in Microcosm 250 (average total fresh biomass 260.6 ± 11.3 g vs. 144.9 ± 14.6 g and average total dry biomass 34.1 ± 2.6 g vs. 13.2 ± 1.4 g, respectively) (Figure 2), as a result of higher weights for all aerial organs apart from stem fresh weights (Figure 3). On the other hand, no significant differences in weights for all the aerial organs were scored in plants grown under 16 and 24 h of lighting (with average total fresh biomasses of 165.5 ± 17.6 and g 178.7 ± 15.9, respectively, and average total dry biomasses of 15.7 ± 2.9 g and 18.3 ± 1.8, respectively) (Figure 4).

### 3.2. CCI Determination

No significant differences in CCI levels were recorded in leaves from Microcosms 250 and 380 (23.6 ± 2.5 and 31.1 ± 3.3, respectively) as well as in leaves from Microcosms 16 and 24 (28.1 ± 1.7 and 35.3 ± 3.3, respectively).

### 3.3. LC-ESI-LTQ-Orbitrap-MS Analysis

Few differences in non-volatile metabolic patterns were scored among samples from Microcosms 250 and 380 and those from samples Microcosms 16 and 24. The metabolic profiling of extracts from apical and middle leaves collected in Microcosms 250 and 380, conducted via LC-ESI-FT-(Orbitrap)-MS and MS/MS analyses, and compared to literature data and spectral data banks of natural products (http://www.massbank.jp/), led to the putative identification of 43 compounds, mainly belonging to flavonoids, phenylpropanoids, organic acids, and catechins, in addition to stilbenes and two triterpene acids (Table 1). The metabolites 3, 9, 13, 31, 33, and 43 were only detected in Microcosm 250 while the metabolites 1, 2, 4, 19, 23, and 27 were only detected in Microcosm 380.

The same metabolite clusters, except for compounds 13, 23, 40, and 43, were detected in apical and middle leaves collected in Microcosms 16 and 24 (Table 2). Metabolites 31 and 33 were only detected in Microcosm 16 whereas metabolites 4, 18, 19, and 27 were only detected in Microcosm 24.

### 3.4. GC-qMS Analysis

Following the HS-SPME GC-MS analysis, 69 volatile compounds belonging to terpenes (53), aldehydes (3), alcohols (8), esters (4), and ketones (1) were identified in leaves from Microcosms 250 and 380. Moreover, 67 VOCs belonging to terpenes (52), aldehydes (3), alcohols (8), esters (2), and ketones (2) were detected in leaves from Microcosms 16 and 24. Almost the same VOCs were observed under the different lighting treatments, even though germacrene D (T38), *cis*-3-hexenyl isovalerate (E3), and methyl benzoate (E4) were not detected in the photoperiod experiment and 1-octen-3-one (O2) was absent in the PPFD experiment. All the detected VOCs are listed in Table 3 and Table 4, in which abbreviation codes, experimental and reported Kovats indexes, and the identification method are also reported.

For each experiment, the HS-SPME/GC-MS semi-quantitative data, calculated as the percentage ratio of the respective peak area to the total peak area of 2-octanone (IS) (RPA%), were subjected to a one-way ANOVA test, using the MetaboAnalyst 5.0 web-based tool, to investigate the effects of the different treatments on the VOCs profiles. The complexity of the data was reduced, as reported in the experimental section. Following the ANOVA test, significant statistical differences in VOC content between the PPFD and the photoperiod experiments were evidenced (*p* < 0.05; Supplementary Tables S1 and S2). Linalool (10–49% of total volatile compounds), eucalyptol (1,8-cineole) (18–45%), and eugenol (6–16%) (Tables S1 and S2) were the main volatile constituents of basil regardless of the lighting conditions.

### 3.5. Multivariate Data Analysis

**Figure 5:**
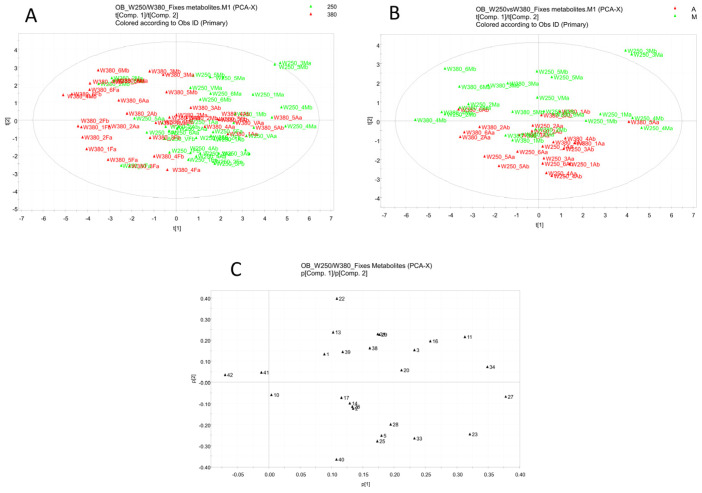
PCA from the LC-ESI-LTQ-Orbitrap-MS pseudo-targeted analysis of non-volatile compounds in leaves from plants in the PPFD experiment. (**A**) The score scatter plot is colored to differentiate between 250 μmol·m^−2^·s^−1^ (250) and 380 μmol·m^−2^·s^−1^ (380). (**B**) The score scatter plot is colored to distinguish apical (A) leaves and middle (M) leaves. (**C**) Loading scatter plot.

To identify volatile and non-volatile compounds that can be used as possible biomarkers associated with the different lighting regimes, a pseudo-targeted approach was developed; this involved building a data matrix that considered and manually took the peak areas of the compounds identified in Table 1 and Table S1 (PPFD experiment) and in Table 2 and Table S2 (photoperiod experiment). The resulting matrices were then subjected to multivariate data analysis using SIMCA®-P+ 12.0 software through the PCA approach. The PCA for non-volatile metabolites in the PPFD experiment (Table 1) is reported in Figure 5.

The score scatter plot (Figure 5A) shows no clear separation of the samples in non-volatile metabolites associated with the different lighting levels of apical and middle leaves. Through a different coloring, a better separation of the middle leaves, located in the upper part of the plot, and the apical leaves, located in the lower part of the plot, is observed (Figure 5B). The loading plot obtained from the PCA highlights marker compounds (Figure 5C) in the leaves collected at different levels in the plant, but not relative to the two PPFD levels. In particular, extracts from apical leaves were characterized by a higher concentration of kaempferol gentiobioside (10), chicoric acid (17), and apigenin (27), whereas the middle leaves were characterized by a higher concentration of dehydrodiisoeugenol (41) and ethanol-2-(dodecyloxy)-1-(hydrogen sulfate) (42).

**Figure 6:**
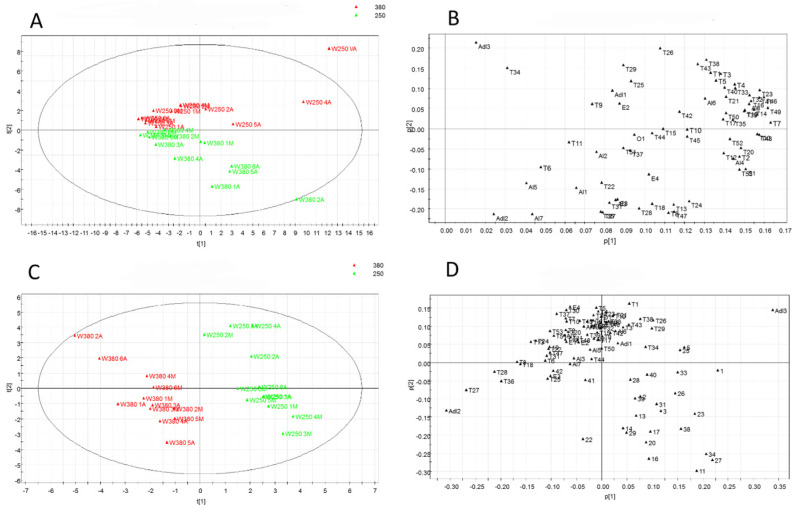
PCA was performed on the GC-MS analysis of volatile compounds in samples from the PPFD experiment and PCA was obtained through data fusion of LC-MS and GC-MS data. (**A**) The volatile compound score scatter plot is colored to differentiate between 250 μmol·m^−2^·s^−1^ (250) and 380 μmol·m^−2^·s^−1^ (380). (**B**) Volatile compound loading scatter plot. (**C**) Data fusion score scatter plot. (**D**) Data fusion loading scatter plot.

Following the multivariate analysis of VOCs obtained by HS-SPME/GC-MS (Table S1, Figure 6), a clear separation of the samples in the PPFD experiment was observed with a similar content of VOCs, which do not separate within the plot. The PCA loading plot (Figure 6B) allows us to highlight individual volatile metabolites (Table S1) that distinctively accumulated under the two PPFD levels. Volatiles detected in the leaves in Microcosm 250 are located in the upper part of the graph, while those detected in the leaves in Microcosm 380 are located in the lower part of the quadrant (Figure 6A). Samples from Microcosm 250 showed a higher number and content of volatiles belonging to the class of sesquiterpenes, including trans-Allocimene (T17), α-Cubebene (T19), α-copaene (T21), β-Cubebene (T23), α-bergamotene (T26), β-elemene (T29), α-humulene (T32), epi-bicyclosesquiphellandrene (T33), trans-β-farnesene (T34), ledene (T35), germacrene D (T38), α-selinene (T39), bicyclogermacrene (T40), α-amorphene (T41), γ-cadinene (T42), β-sesquiphellandrene (T43), α-cadinene (T46), and α-calacorene (T49). Moreover, samples from Microcosm 250 were statistically associated with a higher amount of α-pinene (T1), camphene (T3), β-pinene (T4), sabinene (T5), β-myrcene (T7), D-limonene (T9), *trans*-β-ocimene (T14), α-terpinolene (T16), and bornyl acetate (T25), and, to a lesser extent, two aldehydes, hexanal (Adl1) and *trans*-2-hexanal (Adl3), three alcohols (3-octanol (Al4), 1-octen-3-ol (Al6), and octanol (Al8)), and the ester 1-octen-3-yl-acetate (E2). Conversely, samples from Microcosm 380 were characterized by nine monoterpenes, i.e., α-thujene (T2), α-phellandrene (T6), α-terpinene (T8), β-phellandrene (T10), *cis*-β-ocimene (T12), γ-terpinene (T139, p-cymene (T15), 1,3,8 p-menthatriene (T18), and trans-sabinene hydrate (T20), and six sesquiterpenes, i.e., α-farnesene (T27), α-guaiene (T28), aromadendrene (T30), α-curcumene (T44), Cadina 1,4 diene (T45), and *cis*-Calamenene (T48). Further, samples from Microcosm 380 were directly correlated with three alcohols, i.e., 2-penten-1-ol (Al1), *trans*-2-hexen-1-ol (Al5), and 1-hexanol-2-ethyl (Al7), as well as the aldehyde *cis*-3-hexenal (Adl2), the esters 3-hexen-1-ol-acetate (E1), cis-3-hexenyl isovalerate (E3), and methyl benzoate (E4), and the ketone 3-octanone (O1). Samples from Microcosm 380 were characterized by higher levels of all oxygenated monoterpenes, including 1,8-cineole (T11), camphor (T22), linalool (T24), 4-terpineol (T31), L-borneol (T36), α-terpineol (T37), nerol (T47), β-ionone (T50), methyl eugenol (T51), *trans*-nerolidol (T52), and eugenol (T53). The PCA score scatter plot, which is related to both non-volatile and volatile metabolites (Figure 6C), shows that the samples are separated according to the PPFD level. The left side of the graph shows a higher content of volatile metabolites (4M, 1A, VM), while the right side shows the presence of non-volatile metabolites (3M, 1M, 5M). Therefore, the samples from Microcosm 380 (both middle and apical leaves) displayed a higher content of volatile metabolites, whereas the samples from Microcosm 250 showed a higher content of non-volatile metabolites (Figure 6C,D).

**Figure 7:**
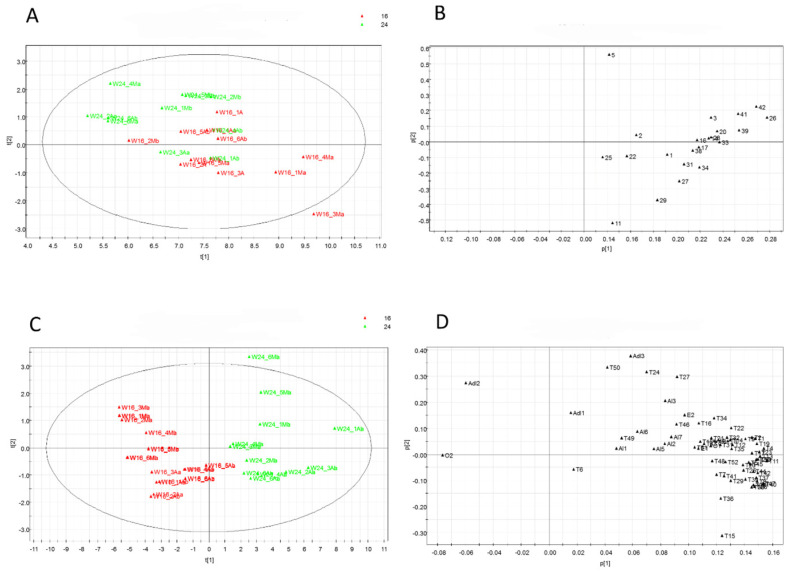
PCA from the LC-ESI-LTQ-Orbitrap-MS pseudo-targeted analysis of non-volatile compounds in samples from the photoperiod experiment; 16, 16/8 h light/dark; 24, 24/0 h light/dark. (**A**) The score scatter plot for non-volatile metabolites. (**B**) The loading scatter plot for non-volatile metabolites. (**C**) Volatile metabolite score scatter plot. (**D**) Volatile metabolite loading scatter plot.

Multivariate data analysis was also conducted to analyze data from the metabolic profiling of plants in the photoperiod experiment. The PCA results, which were obtained following a pseudo-targeted approach on non-volatile metabolites and volatile metabolites, are separately elaborated on and are reported in Figure 7.

The score scatter plot for non-volatile metabolites (Table 2, Figure 7A) highlights a low separation of samples based on the photoperiod length. In particular, samples from Microcosm 16 are positioned in the right lower square, but the different samples do not generate two clusters. In the area displaying the higher presence of samples from plants grown under 16 h of lighting, metabolites belonging to the class of flavonoids and flavonoid glycosides (11, 27, 29), as well as phenolic acids (31, 34), were detected, as shown in the loading scatter plot (Figure 7B). Figure 7C,D show the score scatter plot and the loading scatter plot, respectively, obtained by a PCA performed on the HS-SPME/GC-MS data (Table S2). The first PCA allowed separating the samples into two clusters: samples from Microcosm 16 are located on the left, while samples from Microcosm 24 are located on the right (Figure 7C). The loading scatter plot allows us to identify metabolites responsible for the clusterization of the plants. In particular, on the right of the loading plot, most of the detected VOCs are overexpressed in plants grown in Microcosm 24, while only two volatiles, Adl2 (cis-3-hexenal) and O2 (1-octen-3-one), are overexpressed in the plants grown in Microcosm 16 (Figure 7B and Table S2).

**Figure 8:**
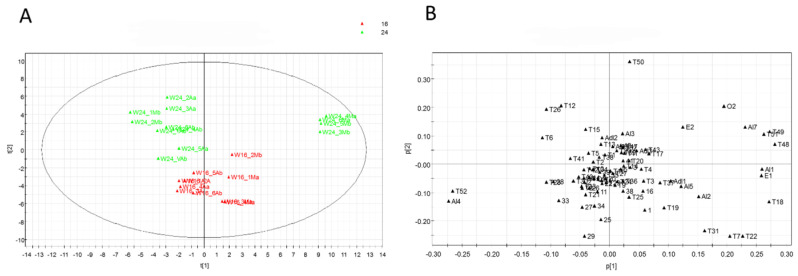
PCA was performed on a data fusion block, combining the results of LC-MS and GC-MS for the analysis of non-volatile and volatile compounds in samples from the photoperiod experiment; 16, 16/8 h light/dark; 24, 24/0 h light/dark. (**A**) Score scatter plot. (**B**) Loading scatter plot.

The score scatter plot and the loading scatter plot, resulting from the combination of variables corresponding to non-volatile and volatile metabolites through a data fusion approach, are reported in Figure 8A,B, respectively.

The clusters of samples were less discriminant compared to the results from the analysis of the non-volatile compounds. In particular, samples from Microcosm 16 are grouped in a single area of the plot defined mainly by the second principal component. Metabolites 27 and 29 (apigenin glucuronide methyl ester and luteolin) are again relevant for the discrimination of samples in the group of non-volatile compounds. With regard to the volatile compounds, samples appear to be classified based on negative markers, with VOCs in lower amounts as relevant compounds for the discrimination (Figure 8B).

## 4. Discussion

Phenotypical development and biomass production in basil grown under artificial lighting are clearly affected by different light spectra [9,12,14,20,25,30,31]. The phenological effects of artificial lighting have been far less investigated, as most of the works dealt with small plants under short cultivation periods, ending before the transition to the flowering stage. In previous investigations with fully expanded basil plants, blue–red light has been reported to be more effective than white light in enhancing plant growth and promoting early flowering when supplied at a higher PPFD [25] but not when white light and blue–red light are supplied at the same PPFD levels [12]. In the present study, an increase in PPFD from 250 to 380 µmol·m^−2^·s^−1^ (+52%) resulted in higher total fresh (+79.8%) and dry (+158.3%) biomass, thus confirming that PPFD is a main driver for biomass production in basil. Interestingly, no significant differences in yield were recorded in plants grown under 16 and 24 h lighting conditions. Different PPFD levels and photoperiods had no effects on phenological development; this suggests that light flux and duration may not be directly involved in the transition from the vegetative to the reproductive stage. No significant differences were also observed in CCI levels, thus indicating that different yields in basil are not necessarily associated with the chlorophyll content. This finding supports the previous hypothesis that plants under long-lasting cultivation periods can adapt their photosynthetic systems to different light regimes, reaching, in this way, similar photosynthetic efficiency at the whole plant level [25].

In addition to primary metabolites, plants produce a wide variety of secondary metabolites, including VOCs, which provide adaptive advantages in relation to the growing environment and can also exert beneficial effects on human health [16,32,33,34,35,36,37,38]. Among the most representative VOCs in sweet basil [26,39,40,41], linalool is characterized by sweeter and floral notes and is reported to be an antioxidant and neuroprotective compound [10]. Eucalyptol, characterized by a spicy and camphor-like odor, is known to be effective against many diseases, including respiratory and digestive disorders, and it has anticancer properties [42]. Eugenol is considered an antioxidant and anticancer molecule [40,43]. In the present work, linalool, eucalyptol, and eugenol were the main volatile constituents of the plants, thus indicating that these molecules accumulate in basil independently from the lighting approach.

It has been proposed that specific light treatments applied during the early development of basil can establish specific metabolic profiles that affect the production of aromatic compounds later on in the biological cycle; once a developmental threshold has been overcome, plants delay the metabolic adaptations to new conditions [10]. Multivariate data analysis of the metabolomics assays aimed to identify volatile and non-volatile compounds that can be used as markers associated with different lighting regimes. The results reported in the present work indicated that non-volatile metabolite patterns were mainly affected by the leaf developmental stage (apical vs. middle) and, to a lesser extent, by light flux and lighting duration. Conversely, a clear separation of samples according to the PPFD level was obtained for the volatile compounds. In particular, plants grown under 250 μmol·m^−2^·s^−1^ accumulated higher sesquiterpene levels, while those grown under 380 μmol·m^−2^·s^−1^ accumulated higher levels of monoterpenes. This finding agrees with the report that light intensity affects the expression of genes coding for enzymes involved in the biochemical pathways of secondary metabolites [7]. The differences in VOC patterns can be related to differences in expression, upregulation, stability, or enzyme activity involved in the biosynthetic pathways of oxygenate monoterpenes (380 µmol·m^−2^·s^−1^) and sesquiterpenes (250 µmol·m^−2^·s^−1^). Since increased stomatal conductance has been reported to be associated with higher PPFD levels [7], a higher concentration of terpenes detected in plants grown under higher PPFD could also be related to a higher stomatal opening and, consequently, a higher rate of terpene evaporation from plants. A phytochrome-based control of genes involved in the synthetic pathways of terpenes has also been reported [44], with sesquiterpene and monoterpene production triggered by far-red and red wavelengths, respectively [45]. In the present work, almost all detected VOCs were overexpressed in plants grown in Microcosm 24. This result is consistent with previous reports, indicating that metabolic processes in plants are modulated not only by the light spectra and flux but also by the photoperiod [15,16]. Most of the VOCs detected in the plants belong to the terpene group and are reported to increase in response to abiotic stresses in a number of plants [44]. Indeed, it has been reported that isoprene, monoterpene, and sesquiterpene emissions increase in response to several abiotic stresses, including exposition to prolonged photoperiods through two possible mechanisms: direct antioxidant effects and membrane stabilization [7,46]. Our results suggest that plants grown under a 24-h lighting photoperiod undergo higher abiotic stress compared to plants grown under a shorter lighting period.

## 5. Conclusions

The present work deals with the effects of different white light supplies on fully expanded basil following long-lasting cultivation, i.e., under typical crop-like conditions. The results indicate that, in addition to spectral variations, increasing the light flux density can improve biomass production and drive the production of key secondary metabolites, mainly volatile compounds, involved in basil quality and biological activity. The results obtained can be useful to better address light supply in indoor basil cultivation because they demonstrate that light flux density is the main driver of biomass production and that variations in VOC profiles can be triggered by light flux density modification. Understanding the effects of PPFD and photoperiods on basil production can also support farmers predict the effect of displacing basil cultivation areas, which is expected to occur due to ongoing climate change for both open-field and greenhouse crops.

## Figures and Tables

**Table 1:** Compounds putatively identified by LC-ESI-Orbitrap-MS and MS/MS numbered in order of elution in extracts of apical (A) and middle (M) leaves from Microcosms 250 (‘M250’) and 380 (‘M380’).

N°	RT	[M-H]^−^	Formula	Δ ppm	Metabolite Identification	M250		M380	
						A	M	A	M
1	5.8	263.0049	C_8_H_7_O_10_	4.7	unknown				
2	5.8	355.1245	C_13_H_23_O_11_	1.9	methyl-glucopyranosyl-glucopyranoside				x
3	6.95	191.0198	C_6_H_7_O_7_	2.6	citric acid	x	x		
4	7.96	593.1497	C_27_H_29_O_15_	0.5	vicenin II			x	x
5	8	417.1040	C_17_H_21_O_12_	1	aloin		x	x	x
6	8.22	197.0454	C_9_H_9_O_5_	3.1	syringic acid	x	x	x	x
7	10.6	353.0872	C_16_H_17_O_9_	1.4	chlorogenic acid	x	x	x	x
8	10.82	193.0505	C_10_H_9_O_4_	5	ferulic acid	x	x	x	x
9	11.08	181.0504	C_9_H_9_O_4_	4.5	homovanillic acid	x	x		
10	12.59	609.1468	C_27_H_29_O_16_	2.9	kaempferol gentiobioside	x	x	x	
11	12.85	447.0982	C_21_H_19_O_11_	2.8	luteolin glucoside	x	x	x	
12	13.55	463.0890	C_21_H_19_O_12_	4.4	quercetin glucoside	x		x	
13	13.6	311.1136	C_15_H_19_O_7_	3.2	glucopyranosyloxy hydroxy allylbenzene	x			
14	13.6	493.1137	C_26_H_21_O_10_	1.5	salvianolic acid A	x		x	x
15	13.81	300.9987	C_14_H_5_O_8_	−0.5	ellagic acid	x			x
16	13.98	665.3887	C_36_H_57_O_11_	1.4	triterpene	x		x	
17	14.15	473.0729	C_22_H_17_O_12_	2.8	chicoric acid	x		x	
18	14.15	475.0869	C_22_H_19_O_12_	−0.3	luteolin glucuronide methyl ester			x	x
19	14.71	339.0723	C_15_H_15_O_9_	2.7	esculin hydrate			x	
20	14.84	431.0982	C_21_H_19_O_10_	2.2	genistin	x	x	x	
21	14.89	717.1475	C_36_H_29_O_16_	1.7	salvianolic acid B		x	x	
22	15.41	359.0775	C_18_H_15_O_8_	1.8	rosmarinic acid	x	x	x	x
23	15.41	445.0776	C_22_H_17_O_11_	3.9	apigenin glucuronide			x	x
24	15.88	203.0827	C_11_H_11_O_2_N_2_	4.5	L-tryptophan		x	x	x
25	16.14	651.2310	C_31_H_39_O_15_	−1.1	epiredisroside A		x	x	x
26	16.36	577.1571	C_27_H_29_O_14_	1.3	rhoifolin		x	x	x
27	16.75	459.0923	C_22_H_19_O_11_	1.2	apigenin glucuronide methyl ester			x	x
28	16.82	327.0873	C_18_H_15_O_6_	3	salvigenin		x	x	x
29	17.48	285.0396	C_15_H_9_O_6_	1.2	luteolin		x	x	x
30	17.52	289.0690	C_15_H_13_O_6_	−5.9	catechin	x	x	x	
31	18.19	501.1038	C_24_H_21_O_12_	0.5	diferuloyl tartaric acid	x			
32	19.35	269.0457	C_15_H_9_O_5_	0.8	apigenin	x		x	
33	19.78	329.0665	C_17_H_13_O_7_	1.3	aurantio-obtusin		x		
34	21.99	373.0922	C_19_H_17_O_8_	1.4	rosmarinic acid methyl ester	x		x	
35	22.6	271.0618	C_15_H_11_O_5_	1.4	naringenin	x		x	x
36	22.6	283.0596	C_16_H_11_O_5_	−1.5	wogonin	x		x	x
37	22.68	343.0223	C_18_H_15_O_8_	4.6	eupatilin		x	x	x
38	23.11	313.0717	C_17_H_13_O_6_	2.6	pectolinarigenin		x	x	x
39	25.23	487.3427	C_30_H_47_O_5_	1.3	asiatic acid	x		x	
40	27.82	291.1997	C_15_H_31_O_3_S	2.8	pentadecanesulfonic acid	x	x	x	
41	29.22	325.1448	C_20_H_21_O_4_	0.2	dehydrodiisoeugenol		x	x	x
42	30.95	471.3459	C_30_H_47_O_4_	−2.2	hederagenin	x	x		x
43	38.83	455.3515	C_30_H_47_O_3_	−0.15	ursolic acid		x		

RT: retention time, Δ ppm: high-resolution mass accuracy.

**Table 2:** Compounds putatively identified by LC-ESI-Orbitrap-MS and MS/MS numbered in order of elution in extracts of apical (A) and middle (M) leaves from Microcosms 16 (‘M16’) and 24 (‘M24’).

N°	RT	[M-H]^−^	Formula	Δ ppm	Metabolite Identification	M16		M24	
						A	M	A	M
1	5.5	263.0049	C_8_H_7_O_10_	-	unknown				
2	5.7	355.1245	C_13_H_23_O_11_	1.9	methyl-glucopyranosyl-glucopyranoside		x		x
3	6.75	191.0198	C_6_H_7_O_7_	2.6	citric acid	x	x	x	x
4	7.98	593.1497	C_27_H_29_O_15_	0.5	vicenin II			x	x
5	8.2	417.1040	C_17_H_21_O_12_	1	aloin	x	x	x	x
6	8.32	197.0454	C_9_H_9_O_5_	3.1	syringic acid	x	x	x	x
7	10.7	353.0872	C_16_H_17_O_9_	1.4	chlorogenic acid	x	x	x	x
8	10.92	193.0505	C_10_H_9_O_4_	5	ferulic acid	x	x	x	x
9	11.18	181.0504	C_9_H_9_O_4_	4.5	homovanillic acid		x		x
10	12.69	609.1468	C_27_H_29_O_16_	2.9	kaempferol gentiobioside	x	x	x	x
11	12.95	447.0982	C_21_H_19_O_11_	2.8	luteolin glucoside	x	x	x	x
12	13.75	463.0890	C_21_H_19_O_12_	4.4	quercetin glucoside	x		x	
13	13.71	311.1136	C_15_H_19_O_7_	3.2	glucopyranosyloxy hydroxy allylbenzene				
14	13.75	493.1137	C_26_H_21_O_10_	1.5	salvianolic acid A	x		x	x
15	13.89	300.9987	C_14_H_5_O_8_	−0.5	ellagic acid	x		x	x
16	14.01	665.3887	C_36_H_57_O_11_	1.4	triterpene	x		x	
17	14.22	473.0729	C_22_H_17_O_12_	2.8	chicoric acid	x		x	
18	14.22	475.0869	C_22_H_19_O_12_	−0.3	luteolin glucuronide methyl ester			x	x
19	14.87	339.0723	C_15_H_15_O_9_	2.7	esculin hydrate			x	x
20	14.98	431.0982	C_21_H_19_O_10_	2.2	genistin	x	x	x	
21	14.99	717.1475	C_36_H_29_O_16_	1.7	salvianolic acid B	x	x	x	x
22	15.66	359.0775	C_18_H_15_O_8_	1.8	rosmarinic acid	x	x	x	x
23	15.71	445.0776	C_22_H_17_O_11_	3.9	apigenin glucuronide				
24	15.98	203.0827	C_11_H_11_O_2_N_2_	4.5	L-tryptophan	x	x	x	x
25	16.45	651.2310	C_31_H_39_O_15_	−1.1	epiredisroside A	x	x	x	x
26	16.56	577.1571	C_27_H_29_O_14_	1.3	rhoifolin	x	x	x	x
27	16.79	459.0923	C_22_H_19_O_11_	1.2	apigenin glucuronide methyl ester			x	x
28	16.88	327.0873	C_18_H_15_O_6_	3	salvigenin	x	x	x	x
29	17.55	285.0396	C_15_H_9_O_6_	1.2	luteolin	x	x	x	x
30	17.77	289.0690	C_15_H_13_O_6_	−5.9	catechin	x	x	x	
31	18.10	501.1038	C_24_H_21_O_12_	0.5	diferuloyl tartaric acid	x			
32	19.38	269.0457	C_15_H_9_O_5_	0.8	apigenin	x		x	
33	19.78	329.0665	C_17_H_13_O_7_	1.3	aurantio-obtusin		x		
34	21.98	373.0922	C_19_H_17_O_8_	1.4	rosmarinic acid methyl ester	x		x	
35	22.61	271.0618	C_15_H_11_O_5_	1.4	naringenin	x		x	x
36	22.65	283.0596	C_16_H_11_O_5_	−1.5	wogonin	x		x	x
37	22.78	343.0223	C_18_H_15_O_8_	4.6	eupatilin		x		x
38	23.13	313.0717	C_17_H_13_O_6_	2.6	pectolinarigenin		x		x
39	25.33	487.3427	C_30_H_47_O_5_	1.3	asiatic acid	x		x	
40	27.99	291.1997	C_15_H_31_O_3_S	2.8	pentadecanesulfonic acid				
41	29.32	325.1448	C_20_H_21_O_4_	0.2	dehydrodiisoeugenol		x		x
42	32.11	471.3459	C_30_H_47_O_4_	−2.2	hederagenin				
43	38.98	455.3515	C_30_H_47_O_3_	−0.15	ursolic acid		x		x

RT: retention time, Δ ppm: high-resolution mass accuracy.

**Table 3:** Volatile organic compounds detected in leaves collected from plants grown in the Microcosms under white light at 250 and 380 μmol·m^−2^·s^−1^ and their identification codes.

Metabolite	Code	^a^ RIt/RIsp	^b^ ID	Metabolite	Code	^a^ RIt/RIsp	^b^ ID
**Terpenes**							
α-Pinene	T1	1026/1021	RI/MS/S	α-Selinene	T39	1700/1688	RI/MS
α-Thujene	T2	1030/1025	RI/MS/S	Bicyclogermacrene	T40	1740/1742	RI/MS
Camphene	T3	1076/1080	RI/MS/S	α-Amorphene	T41	1681/1681	RI/MS
β-Pinene	T4	1106/1118	RI/MS/S	γ-Cadinene	T42	1765/1766	RI/MS
Sabinene	T5	1129/1130	RI/MS/S	β-Sesquiphellandrene	T43	1775/1776	RI/MS
α-Phellandrene	T6	1167/1177	RI/MS/S	α-Curcumene	T44	1784/1784	RI/MS
β-Myrcene	T7	1168/1156	RI/MS/S	Cadina-1,4-diene	T45	1786/1789	RI/MS
α-Terpinene	T8	1180/1186	RI/MS/S	α-Cadinene	T46	1808/1815	RI/MS
Limonene	T9	1198/1206	RI/MS/S	Nerol	T47	1815/1812	RI/MS/S
β-Phellandrene	T10	1202/1202	RI/MS/S	*cis*-Calamenene	T48	1835/1837	RI/MS
1,8-Cineole	T11	1221/1228	RI/MS/S	α-Calacorene	T49	1926/1926	RI/MS
*cis*-α-Ocimene	T12	1251/1245	RI/MS/S	β-Ionone	T50	1963/1964	RI/MS/S
γ-Terpinene	T13	1255/1251	RI/MS/S	Methyl Eugenol	T51	2014/2014	RI/MS/S
*trans*-β-Ocimene	T14	1258/1250	RI/MS/S	*trans*-Nerolidol	T52	2054/2054	RI/MS/S
p-Cymene	T15	1276/1267	RI/MS/S	Eugenol	T53	2141/2141	RI/MS/S
α-Terpinolene	T16	1288/1287	RI/MS/S	**Aldehydes**			
*trans*-Allocimene	T117	1382/1392	RI/MS/S	Hexanal	Ald1	1090/1088	RI/MS/S
1,3,8 p-Menthatriene	T18	1391/1391	RI/MS	*cis*-3-Hexenal	Ald2	1118/1118	RI/MS/S
α-Cubebene	T19	1466/1463	RI/MS	*trans*-2-Hexenal	Ald3	1026/1020	RI/MS/S
*trans*-Sabinene hydrate	T20	1474/1474	RI/MS	**Alcohols**			
α-Copaene	T21	1499/1497	RI/MS	2-Penten-1-ol	Al1	1316/1316	RI/MS/S
Camphor	T22	1521/1518	RI/MS/S	Hexanol	Al2	1363/1360	RI/MS/S
β-Cubebene	T23	1545/1541	RI/MS	*trans*-3-Hexen-1-ol	Al3	1392/1390	RI/MS/S
Linalool	T24	1566/1560	RI/MS/S	3-Octanol	Al4	1404/1396	RI/MS/S
Bornyl acetate	T25	1588/1584	RI/MS/S	*trans*-2-Hexen-1-ol	Al5	1413/1411	RI/MS/S
α-Bergamotene	T26	1598/1590	RI/MS	1-Octen-3-ol	Al6	1464/1465	RI/MS/S
α-Farnesene	T27	1698/1698	RI/MS/S	2-Ethyl-1-hexanol	Al7	1499/1496	RI/MS/S
α-Guaiene	T28	1603/1600	RI/MS	Octanol	Al8	1570/1572	RI/MS/S
β-Elemene	T29	1600/1593	RI/MS/S	**Esters**			
Aromadendrene	T30	1649/1650	RI/MS/S	cis-3-Hexenyl acetate	E1	1321/1321	RI/MS/S
4-Terpineol	T31	1614/1616	RI/MS/S	1-Octen-3-yl-acetate	E2	1389/1379	RI/MS
α-Humulene	T32	1674/1672	RI/MS/S	*cis*-3-Hexenyl isovalerate	E3	1440/1440	RI/MS
Epi-bicyclosesquiphellandrene	T33	1653/1633	RI/MS	Methyl benzoate	E4	1628/1628	RI/MS/S
*trans*-β-Farnesene	T34	1677/1673	RI/MS/S	**Ketones**			
Ledene	T35	1702/1707	RI/MS/S	3-Octanone	O1	1260/1254	RI/MS/S
L-Borneol	T36	1709/1707	RI/MS/S	1-Octen-3-one	O2	1290/1290	RI/MS/S
α-Terpineol	T37	1705/1707	RI/MS/S				
Germacrene D	T38	1716/1716	RI/MS/S				

^a^ RI_t_, Relative retention indexes on polar columns reported in the literature; RI_sp_, relative retention indexes calculated versus n-alkanes (C_8_-C_20_) on the HP-INNOWax column; ^b^ identification method as indicated by the following: RI, Kovats retention index on an HP-INNOWax column; MS, NIST, and Wiley libraries spectra; S, co-injection with authentic standard compounds on the HP-INNOWax column.

**Table 4:** Volatile organic compounds detected in leaves collected from plants grown in Microcosms under 16/8 and 24/0 h light/dark and their identification codes.

Metabolite	Code	^a^ RIt/RIsp	^b^ ID	Metabolite	Code	^a^ RIt/RIsp	^b^ ID
**Terpenes**							
α-Pinene	T1	1026/1021	RI/MS/S	α-Selinene	T38	1700/1688	RI/MS
α-Thujene	T2	1030/1025	RI/MS/S	Bicyclogermacrene	T39	1740/1742	RI/MS
Camphene	T3	1076/1080	RI/MS/S	α-Amorphene	T40	1681/1681	RI/MS
β-Pinene	T4	1106/1118	RI/MS/S	γ-Cadinene	T41	1765/1766	RI/MS
Sabinene	T5	1129/1130	RI/MS/S	β-Sesquiphellandrene	T42	1775/1776	RI/MS
δ-3-Carene	T6	1149/1151	RI/MS/S	α-Curcumene	T43	1784/1784	RI/MS
α-Phellandrene	T7	1167/1177	RI/MS/S	Cadina-1,4-diene	T44	1786/1789	RI/MS
β-Myrcene	T8	1168/1156	RI/MS/S	α-Cadinene	T45	1808/1815	RI/MS
α-Terpinene	T9	1180/1186	RI/MS/S	Nerol	T46	1815/1812	RI/MS/S
D-Limonene	T10	1198/1206	RI/MS/S	*cis*-Calamenene	T47	1835/1837	RI/MS
β-Phellandrene	T11	1202/1202	RI/MS/S	α-Calacorene	T48	1926/1926	RI/MS
1,8-Cineole	T12	1221/1228	RI/MS/S	β-Ionone	T49	1963/1964	RI/MS/S
*cis*-β-Ocimene	T13	1251/1245	RI/MS/S	Methyl Eugenol	T50	2014/2014	RI/MS/S
γ-Terpinene	T14	1255/1251	RI/MS/S	*trans*-Nerolidol	T51	2054/2054	RI/MS/S
*trans*-β-Ocimene	T15	1258/1250	RI/MS/S	Eugenol	T52	2141/2141	RI/MS/S
p-Cymene	T16	1276/1267	RI/MS	**Aldehydes**			
α-Terpinolene	T17	1288/1287	RI/MS/S	Hexanal	Ald1	1090/1088	RI/MS/S
*trans*-Allocimene	T18	1382/1392	RI/MS/S	*cis*-3-Hexenal	Ald2	1118/1118	RI/MS/S
1,3,8 p-Menthatriene	T19	1391/1391	RI/MS	*trans*-2-Hexenal	Ald3	1026/1020	RI/MS/S
α-Cubebene	T20	1466/1463	RI/MS	**Alcohols**			
*trans*-Sabinene hydrate	T21	1474/1474	RI/MS				
*trans*-Linalool oxide	T22	1484/1486	RI/MS	2-Penten-1-ol	Al1	1316/1316	RI/MS/S
α-Copaene	T23	1499/1497	RI/MS	Hexanol	Al2	1363/1360	RI/MS/S
Camphor	T27	1521/1518	RI/MS/S	*trans*-3-Hexen-1-ol	Al3	1392/1390	RI/MS/S
β-Cubebene	T25	1545/1541	RI/MS	3-Octanol	Al4	1404/1396	RI/MS/S
Linalool	T26	1566/1560	RI/MS/S	*trans*-2-Hexen-1-ol	Al5	1413/1411	RI/MS/S
Bornyl acetate	T27	1588/1584	RI/MS/S	1-Octen-3-ol	Al6	1464/1465	RI/MS/S
α-Bergamotene	T28	1598/1590	RI/MS	2-Ethyl-1-hexanol	Al7	1499/1496	RI/MS/S
β-Elemene	T29	1600/1593	RI/MS/S	Octanol	Al8	1570/1572	RI/MS/S
Aromadendrene	T30	1649/1650	RI/MS/S	**Esters**			
4-Terpineol	T31	1614/1616	RI/MS/S	cis-3-Hexenyl acetate	E1	1321/1321	RI/MS/S
α-Humulene	T32	1674/1672	RI/MS/S	1-Octen-3-yl-acetate	E2	1389/1379	RI/MS
Epi-bicyclosesquiphellandrene	T33	1653/1633	RI/MS	**Ketones**			
*trans*-β-Farnesene	T34	1677/1673	RI/MS/S	3-Octanone	O1	1260/1254	RI/MS/S
Ledene	T35	1702/1707	RI/MS/S	1-Octen-3-one	O2	1290/1290	RI/MS/S
L-Borneol	T36	1709/1707	RI/MS/S				
α-Terpineol	T37	1705/1707	RI/MS/S				

^a^ RI_t_, Relative retention indexes on polar columns reported in the literature; RI_sp_, relative retention indexes calculated versus n-alkanes (C_8_-C_20_) on HP-INNOWax column; ^b^ identification method as indicated by the following: RI, Kovats retention index on an HP-INNOWax column; MS, NIST, and Wiley libraries spectra; S, co-injection with authentic standard compounds on the HP-INNOWax column.

## Data Availability

The original contributions presented in the study are included in the article/Supplementary Materials, further inquiries can be directed to the corresponding author.

## Supplementary Materials

The following supporting information can be downloaded at https://www.mdpi.com/article/10.3390/foods13142273/s1, Table S1: VOCs identified by one-way ANOVA and post hoc analysis, found as statistically significant for *p* < 0.05 250 vs. 380; Table S2: VOCs identified by one-way ANOVA and post hoc analysis, found as statistically significant for *p* < 0.05 16 vs. 24.
